# KRCC1, a modulator of the DNA damage response

**DOI:** 10.1093/nar/gkac890

**Published:** 2022-10-16

**Authors:** Fiifi Neizer-Ashun, Shailendra Kumar Dhar Dwivedi, Anindya Dey, Elangovan Thavathiru, William L Berry, Susan Patricia Lees-Miller, Priyabrata Mukherjee, Resham Bhattacharya

**Affiliations:** Department of Cell Biology, University of Oklahoma Health Sciences Center, Oklahoma City, OK 73104, USA; Department of Obstetrics and Gynecology, University of Oklahoma Health Sciences Center, Oklahoma City, OK 73104, USA; Department of Obstetrics and Gynecology, University of Oklahoma Health Sciences Center, Oklahoma City, OK 73104, USA; Department of Obstetrics and Gynecology, University of Oklahoma Health Sciences Center, Oklahoma City, OK 73104, USA; Department of Surgery, University of Oklahoma Health Sciences Center, Oklahoma City, OK 73104, USA; Peggy and Charles Stephenson Cancer Center, University of Oklahoma Health Sciences Center, Oklahoma City, OK 73104, USA; Department of Biochemistry and Molecular Biology, Cumming School of Medicine, University of Calgary, Calgary, Alberta, T2N 4N1, Canada; Peggy and Charles Stephenson Cancer Center, University of Oklahoma Health Sciences Center, Oklahoma City, OK 73104, USA; Department of Pathology, University of Oklahoma Health Sciences Center, Oklahoma City, OK 73104, USA; Department of Cell Biology, University of Oklahoma Health Sciences Center, Oklahoma City, OK 73104, USA; Department of Obstetrics and Gynecology, University of Oklahoma Health Sciences Center, Oklahoma City, OK 73104, USA; Peggy and Charles Stephenson Cancer Center, University of Oklahoma Health Sciences Center, Oklahoma City, OK 73104, USA

## Abstract

The lysine-rich coiled-coil 1 (KRCC1) protein is overexpressed in multiple malignancies, including ovarian cancer, and overexpression correlates with poor overall survival. Despite a potential role in cancer progression, the biology of KRCC1 remains elusive. Here, we characterize the biology of KRCC1 and define its role in the DNA damage response and in cell cycle progression. We demonstrate that KRCC1 associates with the checkpoint kinase 1 (CHK1) upon DNA damage and regulates the CHK1-mediated checkpoint. KRCC1 facilitates RAD51 recombinase foci formation and augments homologous recombination repair. Furthermore, KRCC1 is required for proper S-phase progression and subsequent mitotic entry. Our findings uncover a novel component of the DNA damage response and a potential link between cell cycle, associated damage response and DNA repair.

## INTRODUCTION

Genomic instability is an underlying hallmark of cancer and can arise via multiple mechanisms (1). One of the main mechanisms threatening genomic integrity is DNA damage that is caused by cell intrinsic or extrinsic factors (2). Therefore, cells have developed a series of mechanisms to alleviate damage that help preserve genomic integrity known as the DNA damage response (DDR) (3–5). A critical component of DDR is the presence of checkpoints throughout the cell cycle that ensure that transition from one phase to the next phase occurs under the most optimal conditions (6). The ataxia telangiectasia and Rad-related–checkpoint kinase 1 (ATR–CHK1) axis regulates the intra-S checkpoint that responds to extrinsic sources of DNA damage as well as those arising from replication-associated stress (6,7). Typically, activation of this checkpoint delays firing replication origins and eventually blocks mitotic entry to allow time for efficient DNA repair (5,8). Due to deficiencies in tumor protein 53 and/or retinoblastoma protein 1, deregulation of the G1–S transition is observed in many cancers leading to an increased reliance on the remaining intact DDR pathways, particularly the ATR–CHK1 axis (9,10). Activation of the CHK1-mediated checkpoint is a complex multistep process and begins with ATR-dependent phosphorylation of CHK1 at S317 and S345 following replication stress and associated DNA damage. These initial phosphorylation events trigger autophosphorylation at S296, which facilitates kinase activation (6,11). After CHK1 is activated, it phosphorylates cell division cycle 25A (CDC25A) and targets it for degradation (6,11,12). Although the CHK1 activation, checkpoint maintenance and overall potential as a therapeutic strategy have been investigated, the precise mechanism of CHK1 activation and its impact on cell cycle dynamics remain unclear. Therefore, a better understanding of checkpoint signaling and how it is intertwined with DNA repair and the cell cycle is important from a future cancer therapy perspective.

Little is known about the precise roles of those DDR proteins that link cell cycle, checkpoint activation and DNA repair. We recently introduced the lysine-rich coiled-coil 1 (KRCC1), a protein with unknown biology, as a chromatin enriched nuclear protein that is frequently overexpressed in ovarian cancer and in other malignancies (13). Targeting KRCC1 increased apoptosis and decreased clonal growth and tumor growth *in vivo*. At the molecular level, while CHK2 phosphorylation was unchanged, depleting KRCC1 increased CHK1 phosphorylation (pCHK1-S345) and gamma H2AX (γH2AX) suggestive of enhanced DNA damage. Additionally, phosphorylation of histone 3 at serine 10 (pH3-S10), a mitotic marker, was significantly increased (13). Interestingly, these findings suggested that depleting KRCC1 induced DNA damage and at the same time augmented mitotic progression. It is widely known that the presence of DNA damage activates checkpoint and halts cell cycle. Therefore, we reasoned that the intriguing dichotomy observed after depleting KRCC1 was perhaps due to checkpoint inadequacy.

Here, we aimed to elucidate the biology of KRCC1 and investigate whether it plays a role in the checkpoint maintenance. We report that KRCC1 regulates the CHK1-mediated DDR. Furthermore, KRCC1 inhibition suppresses homologous recombination repair (HRR) and delays S-phase progression resulting in premature mitotic entry. Our findings introduce a novel genome maintenance factor that may be important in the replication stress response and in DNA repair.

## MATERIALS AND METHODS

### Cell culture

OV90, U2OS and HeLa cell lines were purchased from ATCC. DR-GFP U2OS cell line was a kind gift from Dr Jeremy Stark. OV90 cells were routinely cultured in RPMI + 10% FBS; U2OS cells were cultured in McCoy 5A + 10% FBS; and HeLa and DR-GFP U2OS cells were cultured in DMEM high glucose + 10% FBS. All the cells were cultured with 1× penicillin–streptomycin (Gibco, Grand Island, NY, USA) in a 5% CO_2_ humidified atmosphere and tested for mycoplasma contamination prior to any experiment.

### Transfection

Gene silencing was performed in 60 mm culture dish containing 5 × 10^5^ cells in suspension using Lipofectamine RNAiMAX (Thermo Scientific) and 10 picomoles siRNA; scrambled control (SIC001, Sigma) and KRCC1 (SASI-HS01-00181201 and SASI-HS01-00181202, Sigma) in OPTIMEM (Invitrogen). Overexpression was performed using Lipofectamine 3000 (Thermo Scientific) and indicated plasmids in OPTIMEM. WT-CHK1 and CA-CHK1 plasmids were kind gifts from Dr. You-Wei Zhang.

### Cell lysis and western blotting

Total cell lysate was prepared in RIPA (Boston Bioproducts) containing protease and phosphatase inhibitor cocktail (Thermo). The cell lysate was quantified, and proteins separated on an SDS-PAGE gel and transferred to a PVDF membrane (Bio-Rad). Membranes were blocked in 5% nonfat milk in TBS with 0.1% Tween 20 (TBST) for 1 h at room temperature followed by incubation with primary antibodies in TBST with 1% BSA overnight. The following primary antibodies were used: pCHK1-S345 (2348), pCHK1-S296 (2349), γH2AX (2577), CHK1 (2360), HA-tag (3724), pH3-S10 (3377) and pan 14-3-3 (8312) from Cell Signaling Technology (Danvers, MA, USA); KRCC1 (16916-1-AP) from Proteintech (Rosemont, IL, USA); CDC25A (sc-7389) and CDC7 (sc-56275) from Santa Cruz Biotechnology (Dallas, TX, USA); pRPA-S33 (A300-246A), AND-1 (A301-141A), PSF3 (A304-124A), CHK1 (A300-298A) and pMCM2-S40/41 (A300-788A) from Bethyl Laboratories (Montgomery, TX, USA); DBF4 (ab124707) and pCyclinB1-S126 (ab55184) from Abcam; anti-Halo-tag (G921A) from Promega; RPA32 (MABE285) from EMD Millipore; RAD51 (NB100-148) from Novus Biological; 53BP1 (88439) from Cell Signaling Technology; and α-tubulin and β-actin from Sigma–Aldrich. Secondary antibodies conjugated with horseradish peroxidase IgG rabbit (A6154) and mouse (A4416) were obtained from Sigma–Aldrich. Primary antibodies were used in dilutions recommended by the manufacturer. Secondary antibodies were used at a concentration of 1:10 000. Equal loading was verified by immunoblotting with α-tubulin/β-actin.

### Immunofluorescence

OV90 and U2OS cells growing on coverslips were transfected with scrambled (siCTL) or KRCC1 siRNA (siKRCC1). For replication stress evaluation, the cells were fixed followed by immunofluorescence of RPA2 foci formation (Bethyl Laboratories, 1:500). For RAD51 foci formation evaluation, the cells were treated with camptothecin (CPT, 1 µM) for 1 h, washed with fresh warm media and collected after 2 h for the immunofluorescence of RAD51 foci formation (Novus, 1:200). Results were quantified as the percentage of the cells with >10 foci in each treatment. Statistical analysis was performed using Student’s *t* test (*n* = 3). Differences were considered significant at *P* < 0.05.

For RAD51 foci dynamics, OV90 cells were allowed to recover for up to 8 or 24 h and collected at indicated time points for the immunofluorescence of γH2AX (Cell Signaling Technology, 1:300) and RAD51 foci formation, which was quantified as the percentage of the cells with >10 γH2AX and RAD51 foci in each treatment. Statistical analysis was performed using two-way ANOVA (*n* = 3). Differences were considered significant at *P* < 0.05.

### Premature mitotic entry evaluation

HeLa cells growing on coverslips were transfected with scrambled (siCTL) or KRCC1 siRNA (siKRCC1). Cells were pulse labeled with 5-ethynyl-2′-deoxyuridine (EdU) for 15 min, fixed and followed by immunofluorescence of pH3-S10 (Cell Signaling Technology, 1:300) and click reaction for EdU. Statistical analysis was performed using Student’s *t* test (*n* = 3). Differences were considered significant at *P* < 0.05.

### Cell cycle

For cell cycle synchronization in the G1–S phase, a dT block was performed 24 h after siRNA transfections. Cells were treated with 2 mM thymidine (Sigma–Aldrich, St Louis, MO, USA) as illustrated in Figure 4D and collected at the indicated time points. Cells were fixed with 70% ethanol. After washing, the cells were incubated in propidium iodide (PI, F10797; Thermo Fischer Scientific). Ten thousand cells were analyzed using the FACSCalibur flow cytometer (Becton Dickinson, Franklin Lakes, NJ, USA). For EdU/PI cell cycle analysis, asynchronously growing cells were pulse labeled with EdU (20 μM) for 15 min. Cells were fixed and permeabilized followed by detection of EdU by Click-iT (C10424; Thermo Fischer Scientific) and PI for DNA content. Ten thousand cells were analyzed using the FACSCelesta flow cytometer (Becton Dickinson, Franklin Lakes, NJ, USA) and analysis in FlowJo version 10.8.1.

### DNA-mediated chromatin pull down

For analysis of proteins that were associated with nascent DNA, cells were processed and analyzed using the DNA-mediated chromatin pull-down (Dm-ChP) technique, as previously described (14). Briefly, cells were incubated for 30 min with 10 μM EdU, washed with PBS and then cross-linked for 10 min at 4°C by adding 10 ml of 1% (v/v) PFA at room temperature with rocking (40 rpm) prior to quenching with 0.125 M glycine for a further 10 min. Cells were then washed three times with ice-cold PBS, harvested by scrapping and permeabilized with 2 ml of 0.1% (v/v) Triton X-100 in PBS for 10 min on ice and washed with PBS. To perform the Click reaction, the following components were added sequentially: 10 mM (+)-sodium l-ascorbate, 10 μM biotin azide and 2 mM copper(II) sulfate, and cells were incubated in the dark for 30 min at room temperature followed by addition of 1% (w/v) BSA and 0.5% (v/v) Tween 20 in PBS and incubated for a further 10 min. After three washes in PBS, soluble proteins were extracted in 500 μl of CL lysis buffer [50 mM HEPES (pH 7.8), 150 mM NaCl, 0.5% (v/v) NP-40, 0.25% (v/v) Triton X-100, 10% (v/v) glycerol] containing protease inhibitors (Protease Inhibitor Cocktail III, Fisher Scientific, BPE 9709-1) by incubation at 4°C with end-over-end mixing for 10 min followed by slow-speed centrifugation (1300 rpm/150 × *g*). The residual pellet was then washed for 10 min at 4°C by end-over-end mixing in 500 μl of wash buffer [10 mM Tris–HCl (pH 8.0), 200 mM NaCl, 0.5 mM DTT]. The pellet was then resuspended in 500 μl of RIPA buffer containing protease inhibitor cocktail. To shear the chromatin, lysates were sonicated on ice at 40% amplitude for six rounds of 10 s with 2 min interval between rounds using a Digital Sonifier (Branson, UK). The extract was clarified by centrifugation at 16 000 × *g* for 10 min at 4°C. Protein content was quantified using the Pierce BCA Kit (Thermo Scientific, Runcorn, UK) and 25 μg of the supernatant was saved as an input for western blotting analysis. Typically, 1 mg of this extract was used for pull down with 50 μl of wet neutravidin beads. Before use, beads were washed twice with 500 μl wash buffer, equilibrated in RIPA buffer and blocked overnight at 4°C with 0.5 mg/ml BSA and 500 ng pre-sheared empty vector (EV) DNA to minimize nonspecific binding. On the next day, beads were washed three times and transferred to a new tube. Chromatin extracts were incubated for 2–16 h at 4°C with pre-blocked neutravidin beads. After binding, unbound material was collected and beads were washed three times with 500 μl of wash buffer. To reverse protein–DNA cross-linking and elute proteins from neutravidin beads, samples were incubated for 5 min at 95°C in 2× Laemmli sample buffer before immunoblotting.

### Statistics

Statistical analyses of single comparisons were performed using a two-tailed Student’s *t* test. Statistical analyses of multiple comparisons were performed using ordinary one-way or two-way ANOVA with Dunnett correction. Data are represented as mean ± SD using GraphPad Prism version 9.0.0 (GraphPad Software). The analyzed number of samples is indicated in the figure legends. Asterisks indicate significance values as follows: *P < 0.05, **P < 0.01, ***P < 0.001 and ^****^P < 0.0001.

## RESULTS

### KRCC1 regulates the CHK1-mediated checkpoint

**Figure 1.**
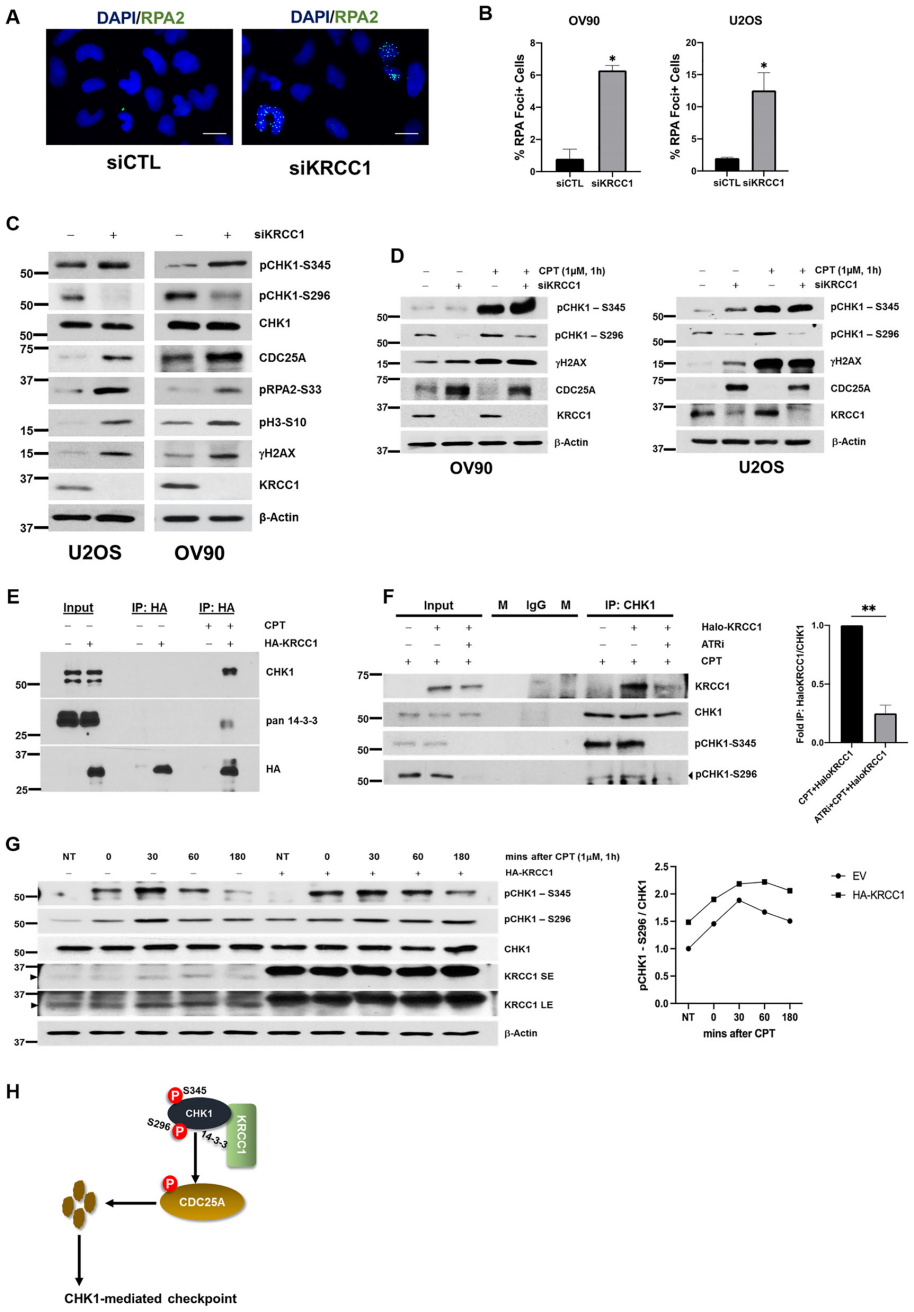
KRCC1 promotes CHK1 activation and efficient checkpoint. (**A**, **B**) OV90 and U2OS cells were transfected with control siRNA (siCTL) or siRNA targeting KRCC1 (siKRCC1) for 72 h. Cells were stained with DAPI and anti-RPA2 antibody and visualized by fluorescence microscopy (scale bar, 20 μm). Percentage of cells with >10 foci is quantitated. The data are from manual scoring of ∼200 cells per condition and from three experiments ± SDs. (**C**) Lysates from above transfected siCTL and siKRCC1 OV90 and U2OS cells were analyzed by immunoblotting for CHK1-mediated DDR markers. (**D**) Immunoblotting for CHK1-mediated DDR markers after 72 h KRCC1 silencing in the presence or absence of CPT (1 μM for 1 h). (**E**) KRCC1 interaction with CHK1 and 14-3-3 was evaluated using co-immunoprecipitation in EV or HA-tagged KRCC1 (HA-KRCC1) overexpressed cells treated with or without CPT (3 μM, 2 h). (**F**) CHK1 interaction with KRCC1 was evaluated using co-immunoprecipitation in EV or Halo-tagged KRCC1 (Halo-KRCC1) overexpressed cells treated with CPT (3 μM, 2 h) in the presence or absence of ATR inhibitor (ATRi, 5 μM, 4 h) and quantification of immunoprecipitated Halo-KRCC1 by densitometry analysis using NIH ImageJ and normalized to their respective CHK1 levels and compared to CPT treatment only, which was set to 1. Experiments were repeated three times. Data represent mean ± SD. Marker (M) and IgG lanes are shown. (**G**) OV90 cells transfected with EV or HA-KRCC1 were treated with CPT (1 μM for 1 h) and released for up to 180 min. Cells were collected at the indicated time points and processed for immunoblotting. The black right-pointing triangle indicates KRCC1. Short exposure (SE) and long exposure (LE) blots for KRCC1 are shown. The right panel depicts quantification of pCHK1-S296 by densitometry analysis using NIH ImageJ, normalized to their respective CHK1 levels and compared to the no treatment control group (NT), which was set to 1. (**H**) Proposed model of CHK1 activation. Following DNA damage and ATR-mediated phosphorylation of CHK1 at S345, CHK1 associates directly or indirectly with KRCC1 and 14-3-3. We posit that this association induces a conformational change in CHK1 to favor autophosphorylation at S296 and enhance kinase activity toward CDC25A.

We previously demonstrated (13) that silencing KRCC1 increased DNA damage while simultaneously increasing pH3-S10, suggesting augmented mitotic progression. Broadly, cell intrinsic DNA damage could be a consequence of replication stress or decreased DNA repair (15). To evaluate replication stress, we measured replication protein A2 (RPA2) foci formation by immunofluorescence and determined the levels of S33 phosphorylation of RPA2 by immunoblotting. RPA2 coats stretches of single-stranded DNA generated during replication stress (15). Silencing KRCC1 in the OV90 ovarian cancer cells and in the U2OS osteosarcoma cells significantly increased RPA2 foci formation by ∼6- and 8-fold, respectively, compared to the control cells (Figure 1A and B). In U2OS and OV90 cells, phosphorylation of RPA2 at S33 also significantly increased (Figure 1C) while total RPA2 did not change (Supplementary Figure S1A), together suggesting enhanced replication stress. ATR is primarily responsible for RPA2-S33 phosphorylation, so these data are consistent with our previous observation (13) that ATR is activated upon KRCC1 inactivation.

Typically, replication stress potentiates an intra-S-phase checkpoint halting cell cycle. However, increased pH3-S10 observed in KRCC1 silenced cells prompted us to evaluate the DDR markers indicative of the integrity of the intra-S checkpoint. To this end, we performed immunoblotting for the DDR markers. We observed that in the KRCC1 silenced cells, γH2AX, CHK1-S345 and pH3-S10 levels were elevated (Figure 1C), while total H3 levels were unchanged (Supplementary Figure S1A). Interestingly, however, although silencing KRCC1 increased DNA damage, CHK1 phosphorylation at S296 decreased and induced stabilization of CDC25A, indicative of a defective checkpoint (Figure 1C). Specificity of the CHK1 phosphorylation antibodies was confirmed by immunoblotting of ATRi, AZD6738, treated cells for CHK1-S345 and CHK1-S296, respectively (Supplementary Figure S1B). Using an independent shRNA in OV90 cells, we further confirmed that silencing KRCC1 increased phosphorylation of CHK1-S345, while it decreased phosphorylation of CHK1-S296 and stabilized CDC25A (Supplementary Figure S1C). To determine that increased phosphorylation of CHK1 at S345 and RPA2 at S33 after KRCC1 silencing was an indication of replication stress and not a result of varying cell cycle distribution, we performed a double thymidine block and released cells into different phases of the cell cycle, which was confirmed by immunoblotting for cyclin E1, cyclin B1 and pH3-S10 (Supplementary Figure S1D). We used CPT, a topoisomerase I inhibitor, treated cells as a positive control for replication stress. We find that cells released into distinct phases after double thymidine block do not show an increase in pRPA2-S33 or pCHK1-S345 but do so after treatment with CPT (Supplementary Figure S1D). Therefore, increased levels of pRPA2-S33 and pCHK1-S345 in KRCC1 silenced cells reflect on replication stress.

To evaluate the impact of KRCC1 on the extrinsic damage-induced checkpoint, we treated control or KRCC1 silenced cells with CPT. Immunoblotting revealed that damage by CPT activated CHK1 and degraded CDC25A to induce checkpoint in control cells (Figure 1D). However, in KRCC1 silenced cells, addition of CPT failed to degrade CDC25A and CHK1-S296 autophosphorylation was reduced despite elevated levels of ATR-dependent CHK1-S345 phosphorylation (Figure 1D). To demonstrate that the observed checkpoint defect was not specific to CPT, we treated control or KRCC1 silenced cells with hydroxyurea (HU) to induce replication stress. Similar to our observations with CPT, treatment with HU increased CHK1 phosphorylation at S345 and S296, and degraded CDC25A in control cells (Supplementary Figure S1E). However, in KRCC1 silenced cells, addition of HU failed to degrade CDC25A and CHK1-S296 autophosphorylation was reduced (Supplementary Figure S1E). In KRCC1 silenced cells, forced expression of wild-type KRCC1 (HA-KRCC1) or exogenous expression of constitutively active CHK1 (CA-MycCHK1) but not wild-type CHK1 (WT-CHK1) led to decreased CDC25A (Supplementary Figure S1F and G). We then tested the effect of re-expression of KRCC1 after CPT treatment. Similar to prior observations, in KRCC1 silenced cells, addition of CPT failed to degrade CDC25A and CHK1-S296 autophosphorylation was reduced compared to CPT alone. However, in KRCC1 re-expressed, CPT treated cells, pCHK1-S296 increased compared to siKRCC1 only and CDC25A was significantly decreased, suggesting rescue of the checkpoint defect (Supplementary Figure S1H).

To investigate an immediate role for KRCC1 in the CHK1-mediated DDR, we determined whether KRCC1 associated with CHK1 by immunoprecipitation. We expressed EV or HA-KRCC1 in OV90 cells and treated with or without CPT. In the presence of DNA damage, we observed association of KRCC1 with CHK1 and with 14-3-3 (Figure 1E). Interestingly, it has been reported that certain isoforms of 14-3-3 associate with CHK1 in a phosphorylation-dependent manner and may be required for phosphorylation and subsequent degradation of CDC25A (12,16,17). To determine whether the association of CHK1 with KRCC1 was dependent on initiating phosphorylation events on CHK1, we expressed Halo-KRCC1 and immunoprecipitated endogenous CHK1 in the presence of CPT and ATRi. Compared to CPT treatment only, the association of KRCC1 with CHK1 decreased upon dual treatment with CPT and ATRi, suggesting that ATR-mediated phosphorylation of CHK1 is important for the association with KRCC1 (Figure 1F). To further investigate how KRCC1 may be regulating CHK1, we expressed EV or HA-KRCC1 in OV90 cells and treated with CPT for 1 h to induce damage, after which cells were washed with fresh media and allowed to recover in media without CPT for up to 180 min. We observed that immediately after CPT treatment until 30 min, CHK1 phosphorylation at S345 and autophosphorylation at S296 increased. However, while autophosphorylation at S296 decreased in the control group at 60 and 180 min, respectively, cells overexpressing KRCC1 sustained phosphorylation even at 180 min (Figure 1G). Altogether, these results suggest that KRCC1 facilitates CHK1 autophosphorylation at S296, which is required for CHK1’s ability to cause CDC25A degradation (Figure 1H).

### KRCC1 impacts homologous recombination repair

**Figure 2.**
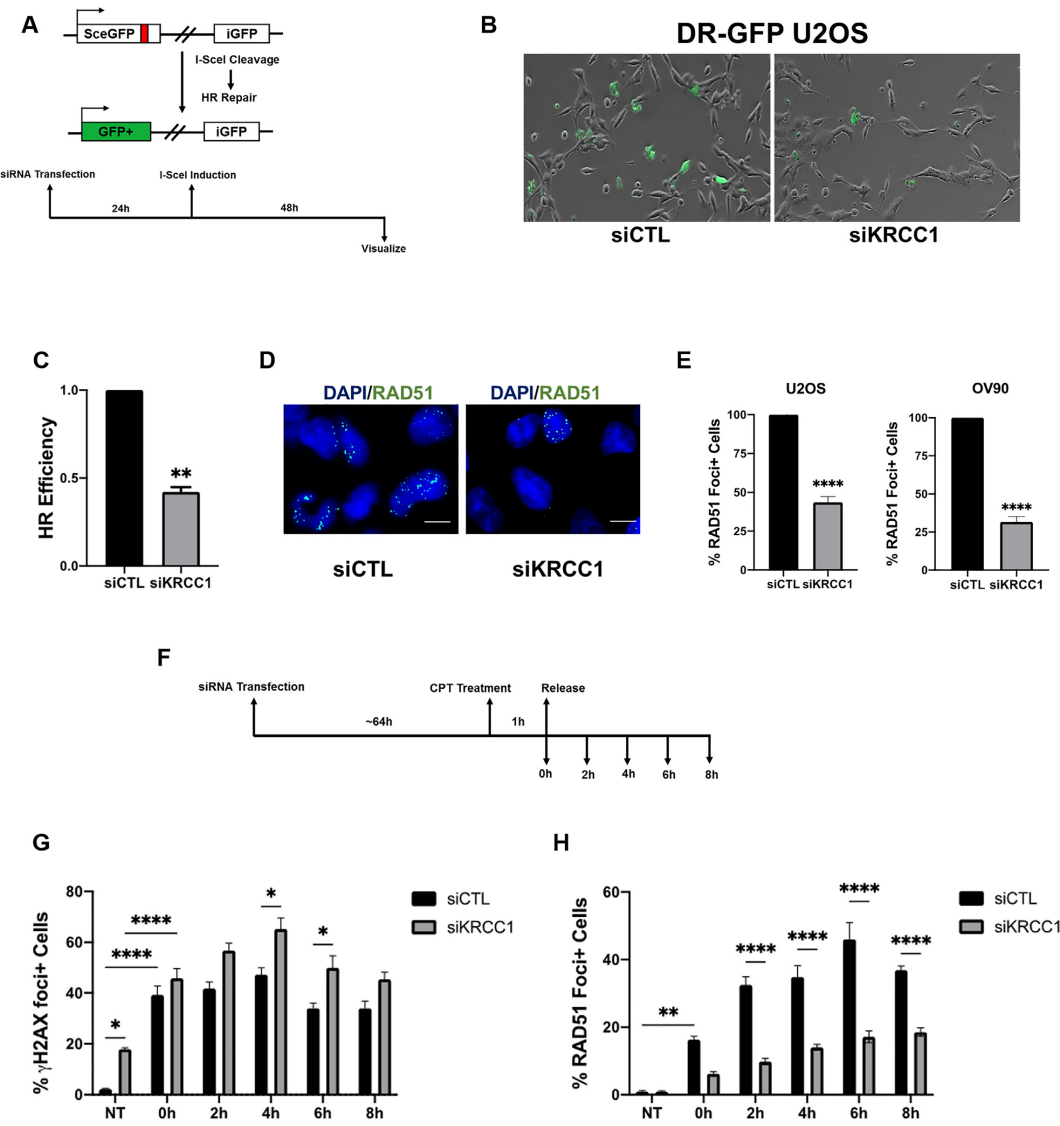
KRCC1 inhibition suppresses HRR. (**A**) Schematic of functional HRR assay. (**B**) Live cell images of DR-GFP U2OS cells transfected with I-SceI endonuclease in the presence or absence of KRCC1 siRNA. (**C**) GFP-positive cells from live imaging were counted and compared to siCTL, which was set to 1. The data are from manual scoring of ∼300 cells per condition per experiment and from three experiments ± SDs. (**D**, **E**) OV90 and U2OS cells transfected with control siRNA (siCTL) or siRNA targeting KRCC1 (siKRCC1) were treated with CPT (1 μM for 1 h) and released for 2 h. Cells were stained with DAPI and anti-RAD51 antibody and visualized by fluorescence microscopy. If a cell had >10 RAD51 foci, it was counted as positive and this was set to 100%. Similarly, in the KRCC1 silenced CPT treated group, RAD51 foci positive cells were counted and expressed relative to the control. Experiments were repeated independently three times and 150 cells were counted per group per experiment. Data represent mean ± SD. (**F**) Experimental design of panels (G) and (H). OV90 cells transfected with control or KRCC1 siRNA and treated with CPT (1 μM for 1 h) were released for 8 h and collected at 2 h intervals. (**G**, **H**) Quantitation of γH2AX and RAD51. Cells with >10 foci were scored as positive. The data are from manual scoring of ∼200 cells per condition per experiment and from three experiments ± SDs.

Checkpoint activation is closely linked to DNA repair. CHK1 specifically has been implicated in the HRR (18,19). To investigate whether KRCC1 silencing impacts HRR, we utilized the well-characterized GFP reporter system DR-GFP in U2OS cells (20). This reporter contains an upstream GFP gene with insertion of an I-SceI recognition site and a downstream internal GFP repeat (iGFP). Introduction of the I-SceI endonuclease induces a double-strand break (DSB) in the upstream GFP. Repair by HRR using the downstream iGFP as the template restores functional GFP expression and the GFP-positive cells can be quantitated (Figure 2A). We measured GFP-positive cells by live cell microscopy. There was a ∼58% decrease in GFP-positive cells after silencing KRCC1, suggestive of a decrease in HRR (Figure 2B and C). In KRCC1 silenced cells, re-expressing KRCC1 significantly increased GFP-positive cells from ∼43% to ∼83%, suggesting a partial rescue of the HRR defect (Supplementary Figure S2A).

To probe the reason for decreased HRR, we performed immunofluorescence in control and KRCC1 silenced cells for RAD51 foci formation, an important step in the HRR pathway (19). RAD51 is a recombinase that directly interacts with the breast cancer-associated tumor suppressor BRCA2 and this interaction is critical for normal recombination proficiency (19,21). Strikingly, there was a ∼60% decrease in RAD51 foci positive cells after depleting KRCC1 (Figure 2D and E) and a ∼58% decrease upon CHK1 inhibition (Supplementary Figure S2B) supporting CHK1 inactivation in KRCC1 silenced cells. We then asked whether the decrease in RAD51 foci reflects delayed or impaired foci formation in KRCC1 silenced cells. To investigate this, we silenced KRCC1 first, then treated with CPT for 1 h and allowed the cells to recover for up to 8 h and determined γH2AX and RAD51 foci formation (Figure 2F). Consistent with immunoblotting results, silencing KRCC1 increased percentage of cells with γH2AX foci in the absence of CPT (Figure 2G). In the presence of CPT, percentage of cells with γH2AX foci increased to ∼34 in control and to ∼45 in KRCC1 knockdown at 8 h indicating robust DNA damage (Figure 2G). After CPT treatment, the percentage of RAD51 foci positive cells continued to increase up to 6 h and a ∼10% decrease was observed at 8 h in control cells (Figure 2H). Although this trend was similar in KRCC1 silenced cells, at all time points the percentage of RAD51 foci positive cells was significantly lower than the controls supporting decreased HRR (Figure 2H, Supplementary Figure S2C). We confirmed by immunoblotting that decreased RAD51 foci was not due to altered expression of RAD51 in KRCC1 silenced cells (Supplementary Figure S2D).

In a similar experiment, we extended the duration of recovery from CPT treatment up to 24 h, to arrive at a phase at which the control cells repaired the damage and resolved γH2AX. As expected, in the control cells 12 and 24 h after CPT treatment the percentage of γH2AX foci positive cells continuously decreased, whereas it remained significantly elevated in the KRCC1 silenced cells (Supplementary Figure S2E). Together, these results suggest that KRCC1 may facilitate efficient recombination and HRR.

### KRCC1 impacts optimal S-phase progression

**Figure 3.**
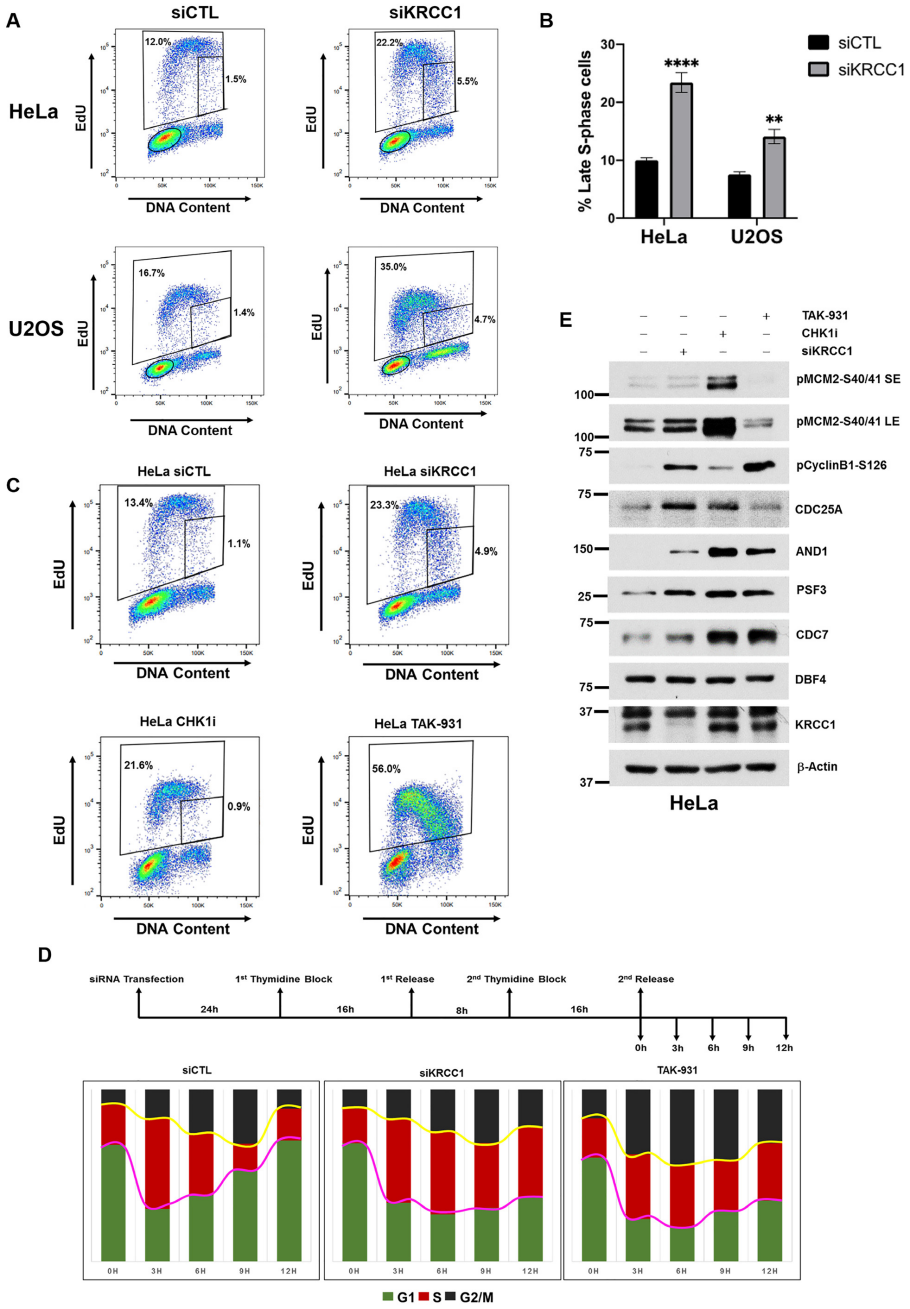
Silencing KRCC1 results in delayed S-phase progression and accumulation of cells at the late S phase. Asynchronous HeLa and U2OS cells transfected with control or KRCC1 siRNA and labeled with 20 μM EdU for 15 min. DNA synthesis, DNA content and cell cycle distribution were assessed by flow cytometry. (**A**) Experimental images of the EdU/PI distribution of control and KRCC1 silenced cells. (**B**) Percentage of cells at the late S–G2 boundary was calculated as a fraction of % EdU-positive cells in the small gate over % total EdU-positive cells. (**C**) HeLa cells transfected with control or KRCC1 siRNA or treated with CHK1i (AZD7762) or CDC7 inhibitor (CDC7i, TAK-931) were labeled with EdU and subjected to flow cytometry. (**D**) HeLa cells transfected with control or KRCC1 siRNA or treated with CDC7i (TAK-931) were G1–S synchronized by double thymidine block and released for 12 h. The cells were collected at the indicated time points and analyzed by flow cytometry. (**E**) Immunoblotting of indicated proteins in control, KRCC1 depleted, CHK1 inhibited or CDC7 inhibited cells. SE and LE blots for pMCM2-S40/41 are shown.

CHK1 plays important roles in the DDR as well as in S-phase progression (7,22); therefore, we assessed whether KRCC1 plays a role in cell cycle progression. In U2OS, OV90 and HeLa cells, we used an EdU/PI flow cytometry assay to surveil cell cycle. We observed that in KRCC1 silenced cells, there was a ∼1.5–2-fold increase in total EdU-positive cells compared to control (Figure 3A). However, relative mean fluorescence intensity of EdU was consistently reduced after silencing KRCC1 (Supplementary Figure S3A and B), suggestive of suboptimal replication (23,24). Interestingly, the percentage of EdU-positive cells at the late S–G2 boundary increased in KRCC1 silenced cells compared to the control (Figure 3A and B, Supplementary Figure S3C), which could be recovered by re-expressing KRCC1 (Supplementary Figure S3D). We reasoned that these changes in the cell cycle dynamics could be due to impaired CHK1 autophosphorylation at S296. To test this, we treated HeLa cells with a CHK1 inhibitor (CHK1i), AZD7762. Like KRCC1 inhibition, the total EdU-positive cells increased; however, the late S-phase accumulation was not observed by CHK1 inhibition (Figure 3C).

Previous reports demonstrated that inhibition of the serine/threonine kinase cell division cycle 7 (CDC7), using a chemical inhibitor TAK-931, leads to stalling at the late S–G2 phases (25), a phenotype similar to what we observed in KRCC1 silenced cells. We then treated HeLa cells with CDC7i, TAK-931. Interestingly, both the total and the percentage of EdU-positive cells at the S–G2 boundary increased phenocopying KRCC1 silencing effects (Figure 3C). To monitor the dynamics of S-phase progression, we performed a double thymidine block time course experiment by flow cytometry. We observed that 3 h after release from the thymidine block, the control cells entered S phase and progressed through S phase by 9 h, reaching the next G1 phase in 12 h (Figure 3D). In contrast, by 3 h although the KRCC1 silenced and the TAK-931 treated cells entered the S phase, progression through S phase was delayed with a significant percentage of cells remaining in the S and G2 phases even after 12 h of release, suggesting that effects of KRCC1 on the cell cycle may be via modulation of CDC7 activity (Figure 3D).

We next asked whether silencing KRCC1 impairs CDC7 activity. Since CDC7 specifically phosphorylates the minichromosome maintenance 2 protein (MCM2) on serine 40 (pMCM2-S40) (25,26), we performed immunoblotting for pMCM2-S40 and related factors involved in replication. Although TAK-931 treatment significantly inhibited pMCM2-S40 phosphorylation and CHK1i treatment significantly increased pMCM2-S40, silencing KRCC1 had no significant impact on pMCM2-S40 phosphorylation status (Figure 3E), despite elevated levels of CDC7, suggesting that CDC7 activity may be impacted by KRCC1 silencing. While CDC25A was stabilized in KRCC1 silenced and CHK1i treated cells, TAK-931 treated cells showed no change, consistent with our observation that KRCC1 silencing impairs CHK1 activity. We then evaluated CDK1 activity by immunoblotting for pCyclinB1-S126. Cyclin-dependent kinase 1 (CDK1) has prominent roles in checkpoint maintenance and in DNA replication (27). Interestingly, silencing KRCC1 increased pCyclinB1-S126 indicating enhanced CDK1 activity. While it is possible that increased activity of CDK1 in KRCC1 depleted cells is a result of CDC25A stabilization, TAK-931 treated cells showed a similar increase in CDK1 activity despite no change in CDC25A status. This suggests that in KRCC1 silenced cells, increased CDK1 activity may not be a CHK1 inhibition effect; in fact, CHK1i treated cells show only a moderate increase in pCyclinB1-S126 (Figure 3E). Additionally, protein levels of the acidic nucleoplasmic DNA-binding protein 1, which is required for recruitment of DNA polymerase alpha (28), increased upon silencing KRCC1 and upon inhibition of CDC7 or CHK1, respectively. PSF3 (GINS complex subunit 3), a component of the replicative helicase (29), and CDC7 increased after CHK1i treatment, TAK-931 treatment and with silencing KRCC1, while DBF4 zinc finger remained unchanged (Figure 3E). Altogether, these results corroborate a role for KRCC1 in mediating checkpoint and suggest a distinct potential for a role in DNA replication during unperturbed cell cycle.

### KRCC1 facilitates efficient mitotic entry

**Figure 4.**
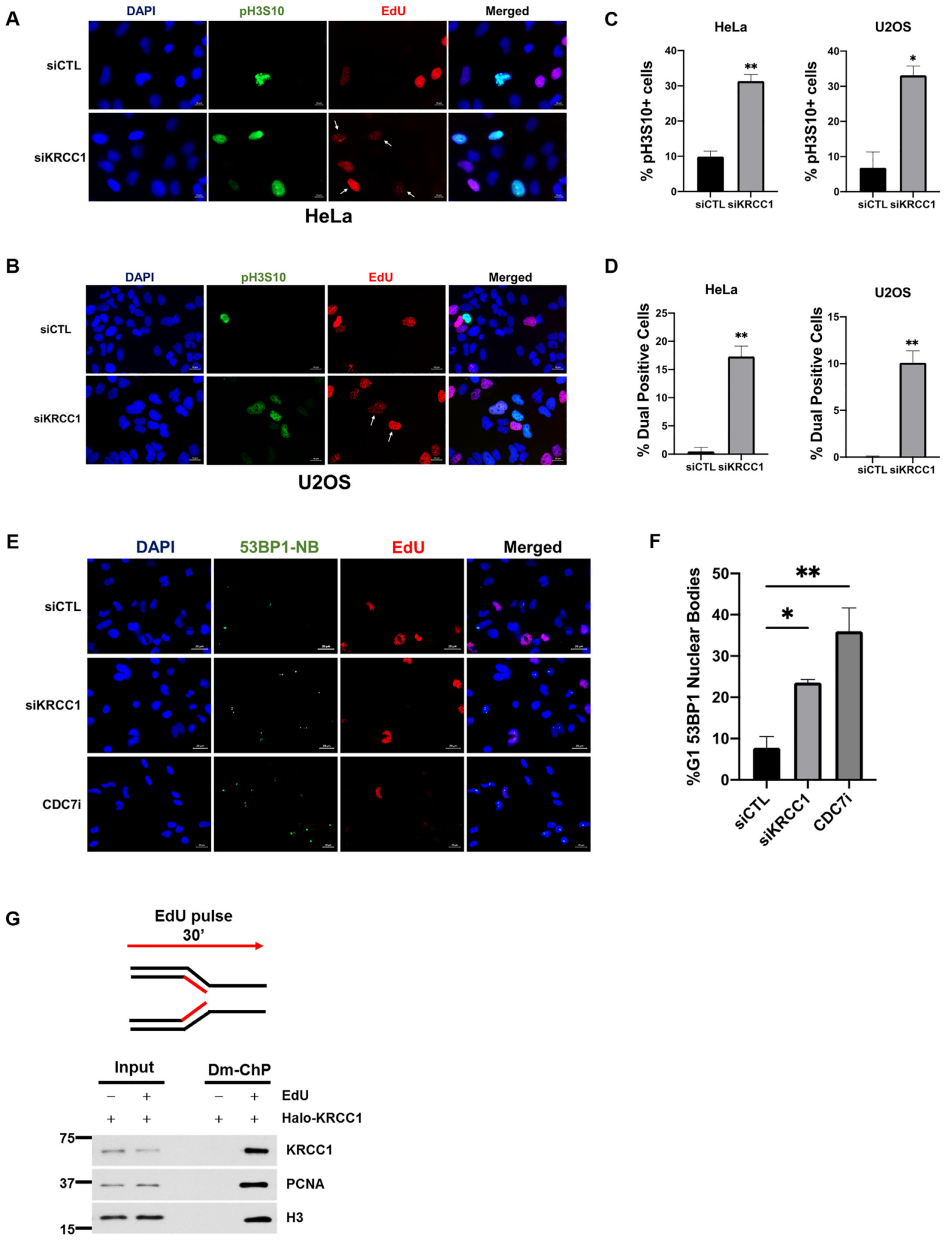
KRCC1 depletion results in premature mitotic entry. (**A**, **B**) HeLa and U2OS cells transfected with control or KRCC1 siRNA and labeled with 20 μM EdU for 15 min. Cells were stained with DAPI and then immunofluorescence was performed for EdU and pH3-S10. (**C**) Percentage of pH3-S10 positive cells. (**D**) Percentage of EdU and pH3-S10 dual positive cells. The data shown are from three independent experiments ± SDs. (**E**) HeLa cells were transfected with control or KRCC1 siRNA or treated with CDC7i, TAK-931 (300 nM, 24 h). The cells were pulse labeled with EdU for 15 min, fixed and processed for immunofluorescence for EdU to denote S-phase cells and 53BP1 to label under-replicated DNA sequestered in 53BP1 nuclear bodies (53BP1-NBs). (**F**) Percentage of G1 cells with 53BP1-NBs. Experiments were repeated independently at least three times. Data represent mean ± SD; ordinary one-way ANOVA was performed for statistical analysis and asterisks indicate significance. (**G**) HeLa cells were labeled with EdU for 30 min, and then cells were fixed and proteins binding to EdU-labeled DNA captured by the Dm-ChP technique as described in the ‘Materials and Methods’ section.

We considered that an impaired intra-S and G2/M checkpoint and delayed S-phase progression, coupled with an increase in pH3-S10, may indicate premature mitotic entry; that is, cells that have not fully replicated their genome (EdU+) would also be positive for the mitotic marker, pH3-S10. To probe this phenomenon, we pulse labeled control and KRCC1 silenced cells with EdU after which cells were fixed and processed for immunofluorescence (Figure 4A and B). Supporting immunoblotting results, there was a significant increase in the percentage of pH3-S10 positive cells after silencing KRCC1 in HeLa or U2OS cells (Figure 4C). Strikingly, ∼10–15% of the KRCC1 silenced cells were dual positive for EdU and pH3-S10, which was not observed in control cells (Figure 4A, B and D).

To determine whether increased premature mitotic entry is a consequence of CHK1 inhibition or CDC7 inhibition, we evaluated EdU and pH3-S10 upon CHK1 inhibition alone (CHK1i), CDC7 inhibition alone (CDC7i) and dual inhibition of CHK1 and CDC7 (CHK1i/CDC7i) in HeLa cells. Compared to control cells, we observed that upon CHK1 inhibition alone, there was a ∼10% increase in pH3-S10 positive and ∼6% increase in EdU and pH3-S10 dual positive cells. Upon CDC7 inhibition alone, we observed a ∼28% increase in pH3-S10 positive and ∼16% increase in EdU and pH3-S10 dual positive cells. Interestingly, upon dual inhibition of CHK1 and CDC7, we observed a ∼22% increase in pH3-S10 positive and ∼21% increase in EdU and pH3-S10 dual positive cells (Supplementary Figure S4A and B). Our results are supported by prior studies that demonstrate that dual inhibition of the ATR–CHK1 axis and CDC7 results in unscheduled mitotic progression with partially replicated DNA (30).

To further confirm this, we investigated the presence of under-replicated DNA in KRCC1 silenced cells. The under-replicated DNA is sequestered in G1 nuclear compartments called 53BP1-NBs (31). These are distinct from DSB-induced 53BP1 foci that are much smaller in size and exclusive to the S phase (31,32). We pulse labeled control, KRCC1 silenced and CDC7i treated cells with EdU. Cells were fixed and processed for immunofluorescence for 53BP1. We labeled with EdU to denote S phase and exclude 53BP1 foci. We observed that compared to the control, KRCC1 silenced cells showed a significant ∼16% increase in G1 53BP1-NBs, while CDC7i treated cells showed a significant ∼28% increase in G1 53BP1-NBs (Figure 4E and F).

The presence of under-replicated DNA prompted us to hypothesize that KRCC1 may act at replication forks. Therefore, we performed the Dm-ChP (14) to assess whether KRCC1 could be specifically captured on nascent DNA. We observed that, similar to the positive control proliferating cell nuclear antigen, KRCC1 could be efficiently cross-linked to newly synthesized DNA (Figure 4G), suggesting that it localizes to replication forks and may be playing a role in the replication machinery.

Together, these results highlight an importance of KRCC1 in mitotic entry and suggest a role in replication.

## DISCUSSION

**Figure 5.**
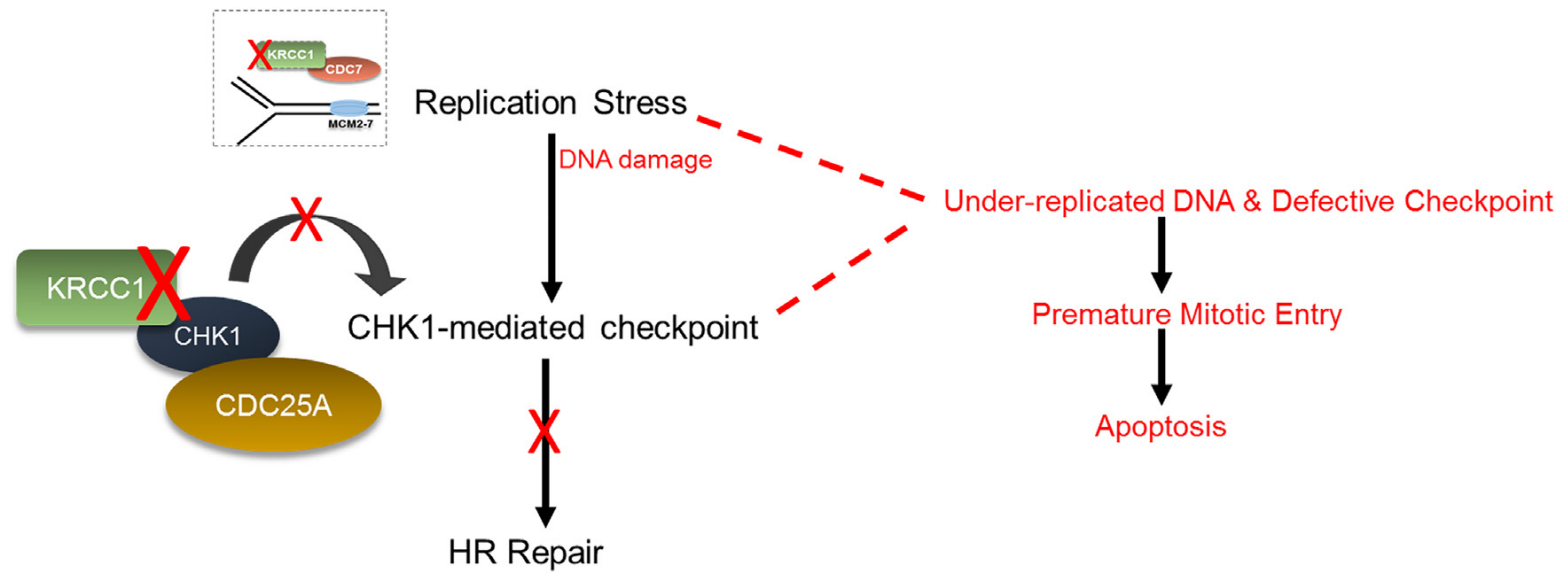
Model of the role of KRCC1 in genome maintenance. Following replication stress and DNA damage, ATR phosphorylates CHK1 at S345. KRCC1 then associates directly or indirectly with CHK1 and 14-3-3 to promote autophosphorylation of CHK1 at S296 and facilitate kinase activity toward CDC25A. CDC25A is then targeted for proteasomal degradation to induce a CHK1-mediated checkpoint. Failure to fully activate CHK1 may result in reduced HRR. We speculate that the KRCC1–CDC7 axis may promote CDC7-mediated events and overall replication integrity, disruption of which leads to replication stress. Overall, replication defects and failure to fully activate checkpoint may result in premature mitotic entry and subsequent apoptosis.

In an effort to elucidate the biology of KRCC1, we uncovered a key modulatory component of the DDR. Based on our results, we posit that upon replication stress and DNA damage, ATR phosphorylates CHK1 at S345. KRCC1 then associates directly or indirectly with CHK1 and 14-3-3 to promote autophosphorylation of CHK1 at S296 and facilitate kinase activity toward CDC25A to induce a CHK1-mediated checkpoint. Failure to fully activate CHK1 may result in reduced HRR. We speculate that the KRCC1–CDC7 axis may promote CDC7-mediated events and overall replication integrity, disruption of which leads to replication stress. Overall, checkpoint inadequacy and replication defects lead to premature mitotic entry (Figure 5) and subsequent apoptosis as we previously reported (13).

Activation of CHK1 requires initial phosphorylation on ATR sites (S345 and S317) followed by autophosphorylation at S296, which expands CHK1 signals (6). This is an important step in the overall activation of checkpoint because it favors interaction with 14-3-3γ and allows phosphorylation of CDC25A leading to its degradation (12,16). Although CHK1 phosphorylation at S345 is sustained, autophosphorylation at S296 is decreased in KRCC1 silenced cells leading to stabilization of CDC25A, ultimately impairing checkpoint activation. Consistent with previous reports, some chemical inhibitors of CHK1 (i.e. AZD7762) also increase phosphorylation of CHK1 at S345 and decrease CHK1-S296 leading to inhibition of CDC25A degradation (18,33). We observe that CHK1 associates with KRCC1 in an ATR-dependent manner. Also, re-expression of KRCC1 in silenced CPT treated cells restores pCHK1-S296 to near CPT treated control levels. We therefore speculate that association with KRCC1 induces a conformational change in CHK1 favoring autophosphorylation at S296 and kinase activity toward CDC25A. Reportedly, CHK1 directly interacts with and phosphorylates RAD51 on T309, which is required for efficient recombination and repair (19). Therefore, in KRCC1 silenced cells, decreased HRR may be due to reduced RAD51 phosphorylation and foci formation, a consequence of impaired CHK1 activity. However, we cannot exclude impaired end resection or BRCA–PALB2 complex formation as possible reasons for decreased HRR in KRCC1 silenced cells.

We find that KRCC1 localizes to replication sites during unperturbed cell cycle and silencing KRCC1 caused replication stress and delayed S-phase progression that leads to accumulation of cells at the late S phase. However, while inhibition of CHK1 did not, inhibition of CDC7 delayed S-phase progression and accumulated cells at the late S phase. During the G1 phase, MCM2–7 heterohexamers are recruited onto potential origins by the cell division cycle 6 and chromatin licensing and DNA replication factor 1 (34). CDC7, a serine/threonine kinase, is then activated during the late G1/early S phase after binding to its regulatory protein, DBF4. CDC7 then phosphorylates MCM2 on S40 to initiate DNA synthesis. CDC7 also plays important roles in the DNA replication fork maintenance by coordinating MRE11-dependent processes on stalled forks, independently of its role in origin firing (35) as well as auxiliary roles in DDR (34). Interestingly, in CDC7 inhibited cells, phosphorylation of MCM2 and origin firing decrease resulting in fewer active forks available to complete DNA synthesis. This increases fork pausing time when obstacles are encountered, thereby extending the length of the S phase (30).

ATR/CHK1 inhibition, on the other hand, increases phosphorylation of MCM2 and origin firing (36,37). However, inhibition of ATR/CHK1 following CDC7 inhibition results in a burst of origin firing in a CDK1- and CDC7-dependent manner (30). The changes in MCM2 phosphorylation following ATR/CHK1 or CDC7 inhibition alone are normalized to near control levels upon dual inhibition (37). Interestingly, while silencing KRCC1 did not impact MCM2 phosphorylation significantly, CDK1 activity was increased, evidenced by increased pCyclinB1-S126. Consistent with the literature that dual inhibition of CDC7 and ATR/CHK1 drives cells into a premature and defective mitosis (30), KRCC1 silenced cells exhibit premature mitotic entry. This is likely due to the accumulation of under-replicated DNA and impaired checkpoints (30,36). We conclude that KRCC1 may support replication fork integrity to ensure efficient DNA replication during unperturbed cell cycle and facilitate optimal checkpoint activation upon replication stress and DNA damage.

Future studies will focus on understanding how KRCC1 impacts the overall integrity of DNA replication.

## DATA AVAILABILITY

FlowRepository IDs FR-FCM-Z59L, FR-FCM-Z59M and FR-FCM-Z59N (http://flowrepository.org/).

## Supplementary Material

gkac890_Supplemental_Files
