# Supplementary material for: KRCC1, a modulator of the DNA damage response
Source: Nucleic Acids Res. 2022 Oct 16;50(19):11028–39. doi: 10.1093/nar/gkac890 (PMC9638924; doi:10.1093/nar/gkac890)
Supplement: gkac890_Supplemental_Files [file gkac890_supplemental_files.zip › Supplementary Figure Legends_R2.docx]

**Supplemental Figure S1 KRCC1 regulates the CHK1-mediated checkpoint.**

**A.** OV90 and U2OS cells were transfected with control siRNA (siCTL) or siRNA targeting KRCC1 (siKRCC1) were analyzed by immunoblotting for RPA2 and H3.

**B.** OV90 cells treated with or without CPT (1μM for 1hr) and ATR inhibitor, AZD6738 (ATRi, 5μM for 4hr) were immunoblotted for pCHK1-S345 and pCHK1-S296.

**C.** Stable OV90 cell lines expressing sh-RNA targeting KRCC1 (shKRCC1) or non-target shRNA (shCTL) were immunoblotted for markers of CHK1-mediated DDR.

**D.** HeLa cells synchronized in the G1/S phase by double thymidine block were released into S- and G2/M phases and collected for immunoblotting. Concurrently, asynchronously growing cells were treated with or without CPT and analyzed by immunoblotting for DDR and cell cycle markers.

**E.** Immunoblotting for markers of CHK1 mediated DDR markers after 72hrs of KRCC1 silencing in the presence or absence of hydroxyurea (HU, 2mM for 2hrs).

**F.** OV90 cells were transfected with 0.5μg, 0.75μg, or 1.5μg of HAKRCC1 following control or KRCC1 depletion by siRNA.

**G.** OV90 cells were transfected with control siRNA (siCTL) and treated with or without CPT (1μM for 1hr) or transfected with WT-CHK1, CA-CHK1, or 1.5μg of HA-KRCC1 following KRCC1 depletion by siRNA were analyzed by immunoblotting for DDR markers. Overexpressed, exogenous (Exo) and endogenous (Endo) proteins shown; indicates KRCC1.

**H.** OV90 cells were transfected with control siRNA (siCTL) and treated with or without CPT (1μM for 1hr) or transfected with KRCC1 siRNA only (siKRCC1) or with 1.5μg of HA-KRCC1 following KRCC1 depletion by siRNA and treated with CPT were analyzed by immunoblotting for DDR markers. indicates KRCC1. Short exposure (SE) and long exposure (LE) blots for KRCC1 are shown.

**Supplemental Figure S2 Silencing KRCC1 inhibits RAD51 foci formation resulting in unrepaired DNA damage.**

**A.** Quantitation of GFP-positivity in cells transfected with siKRCC1 only or HA-KRCC1 following KRCC1 depletion (siKRCC1/HA-KRCC1) and compared to control group (siCTL) which was set to 1.

**B.** Quantitation of RAD51 foci positivity in cells transfected with control or KRCC1 siRNA or treated with CHK1 inhibitor, AZD7762 (CHK1i, 1μM for 4hr). Cells were treated with CPT (1μM for 1hr) and released for 2hrs.

**C.** Immunofluorescence images of OV90 cells transfected with control or KRCC1 siRNA and treated with CPT (1μM for 1hr) were released for 8hrs and collected at 2hr intervals.

**D.** Immunoblotting for RAD51 in control or KRCC1 siRNA transfected OV90 cells treated with CPT (1μM for 1hr).

**E.** Quantitation of γH2AX from immunofluorescence of OV90 cells transfected with control or KRCC1 siRNA and treated with CPT (1μM for 1hr) were released for 24hrs and collected at indicated timepoints. Cells with >10 foci were scored as positive.

**Supplemental Figure S3 KRCC1 promotes optimal S-phase progression.**

**A, B.** Relative EdU intensities of HeLa, U2OS, and OV90 cells transfected with control or KRCC1 siRNA and labeled with EdU for 15mins and assessed by flow cytometry.

**C.** Percentage of OV90 cells at the late S-G2 boundary in control and KRCC1 siRNA transfected conditions.

**D.** Aysnchronous HeLa cells transfected with control or KRCC1 siRNA or with HA-KRCC1 following KRCC1 depletion were labeled with EdU for 15mins and assessed by flow cytometry for cell cycle distribution.

**Supplemental Figure S4 Dual inhibition of CDC7 and CHK1 exacerbates premature mitotic entry.**

**A.** Asynchronously growing HeLa cells were treated with CHK1 inhibitor only (200nM, CHK1i), CDC7 inhibitor only (300nM, CDC7i), or both CHK1 and CDC7 inhibitors (CHK1i/CDC7i) for 24hrs and labeled with 20μM EdU for 15mins. Cells were stained with DAPI following immunofluorescence was performed for EdU and pH3S10.

**B.** Percentage of pH3S10 positive cells.

**C.** Percentage of EdU and pH3S10 dual positive cells. The data shown are from 3 independent experiments ± SDs.
