# Supplementary figures and images for: KRCC1, a modulator of the DNA damage response
Source: Nucleic Acids Res. 2022 Oct 16;50(19):11028–39. doi: 10.1093/nar/gkac890 (PMC9638924; doi:10.1093/nar/gkac890)

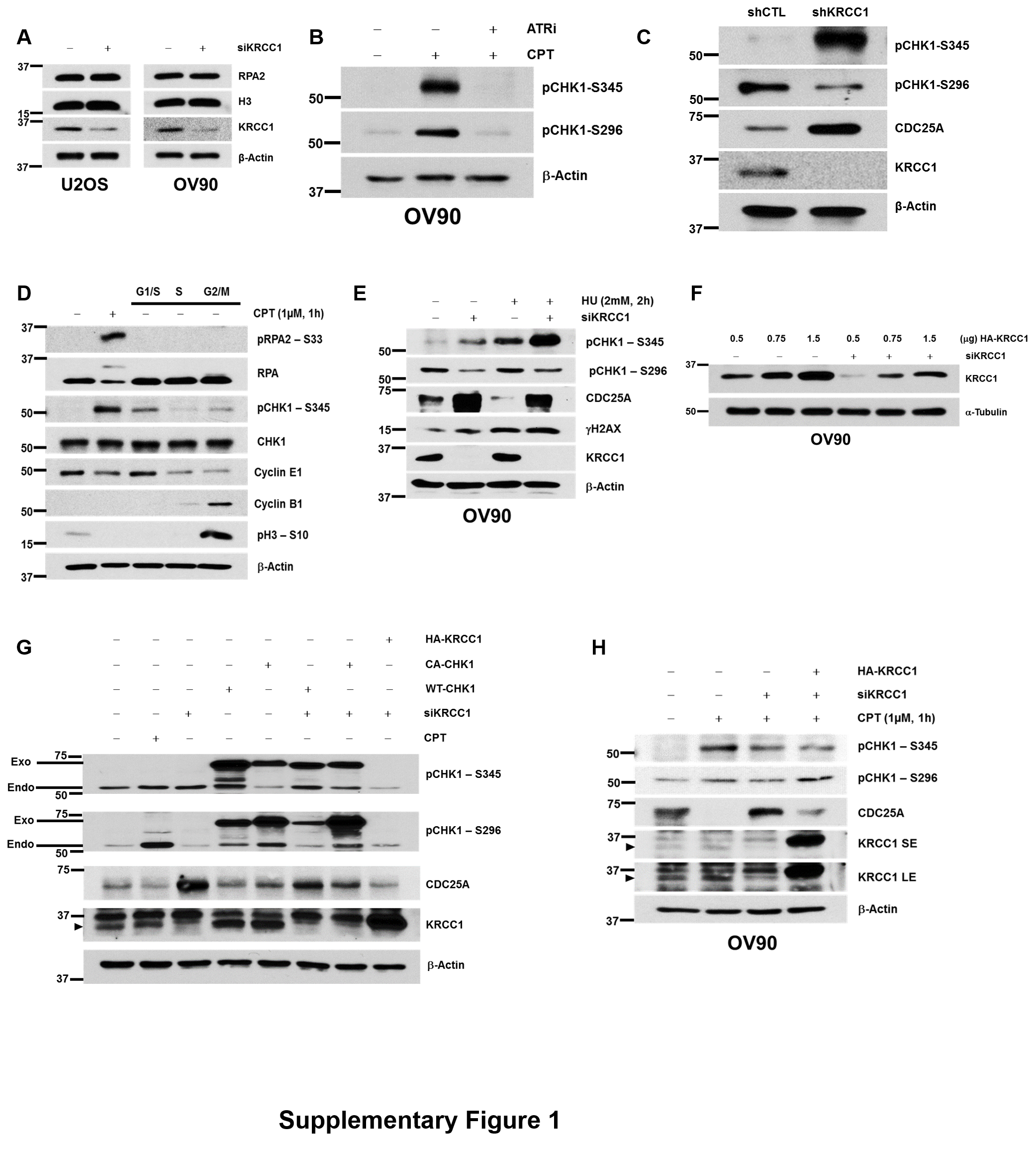

Supplement: gkac890_Supplemental_Files [file gkac890_supplemental_files.zip › Supplemental Figure 1.tif]

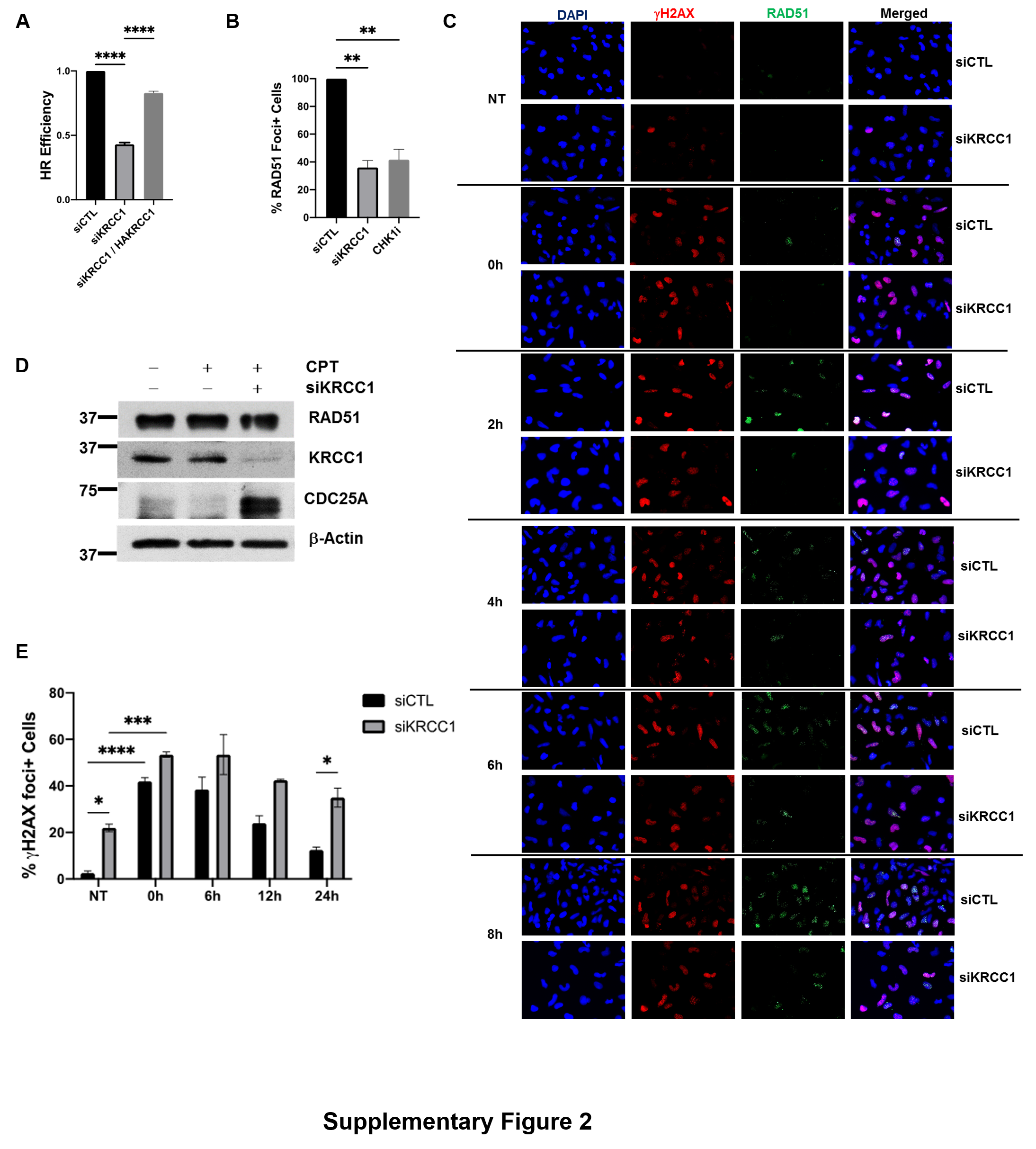

Supplement: gkac890_Supplemental_Files [file gkac890_supplemental_files.zip › Supplemental Figure 2.tif]

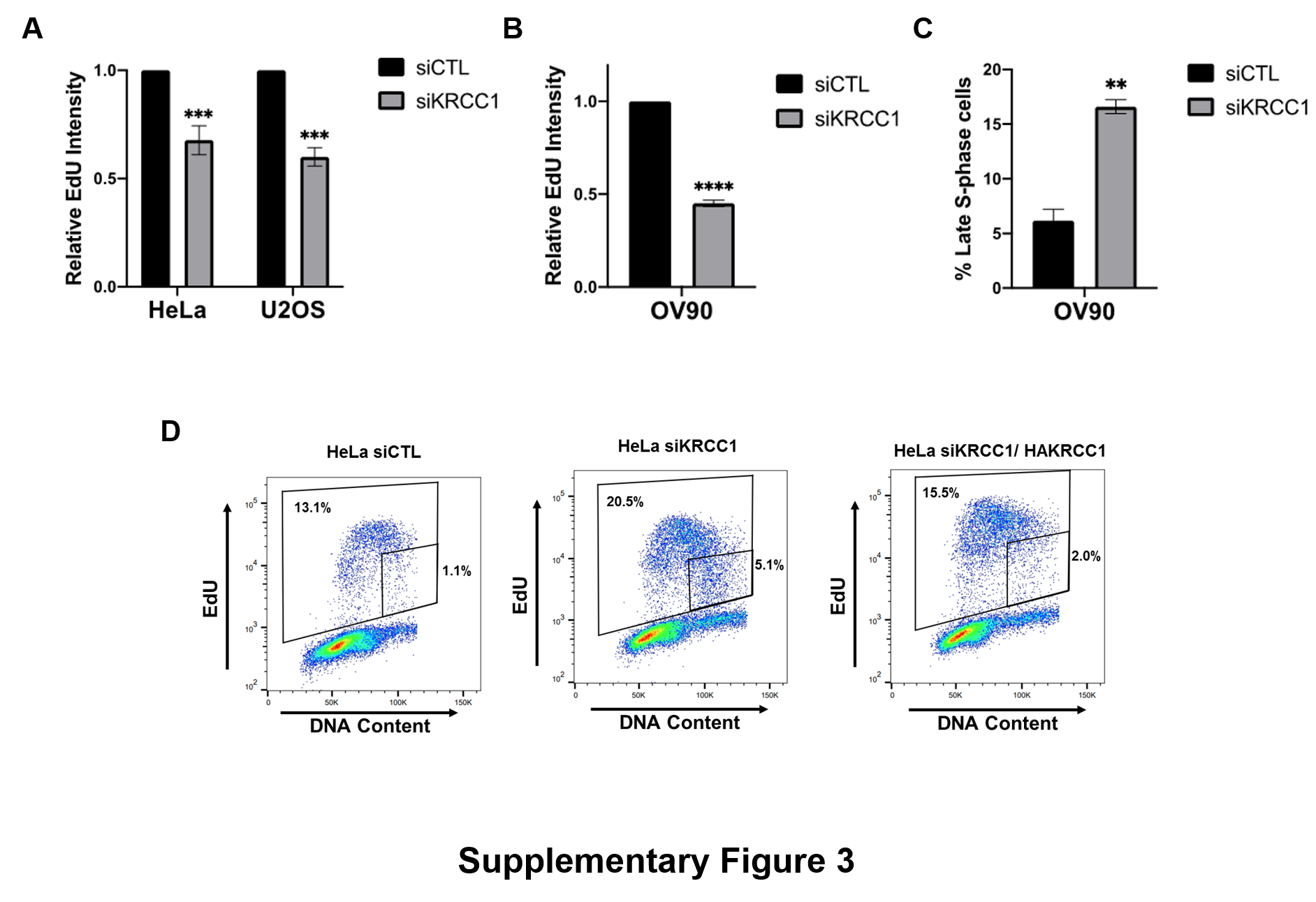

Supplement: gkac890_Supplemental_Files [file gkac890_supplemental_files.zip › Supplemental Figure 3.tif]

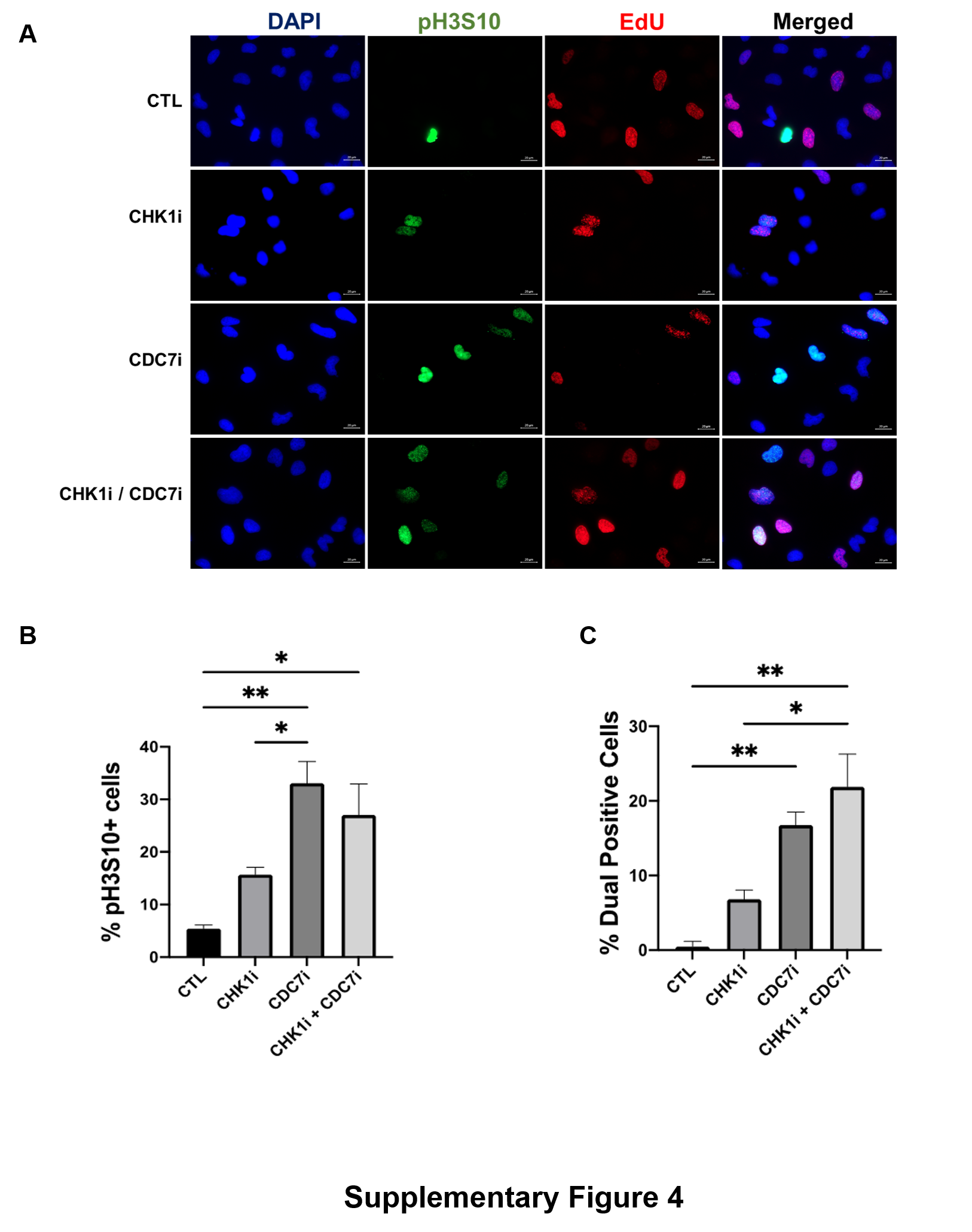

Supplement: gkac890_Supplemental_Files [file gkac890_supplemental_files.zip › Supplemental Figure 4.tif]
